# Nitrogen enrichment impacts on boreal litter decomposition are driven by changes in soil microbiota rather than litter quality

**DOI:** 10.1038/s41598-017-04523-w

**Published:** 2017-06-22

**Authors:** Nadia I. Maaroufi, Annika Nordin, Kristin Palmqvist, Michael J. Gundale

**Affiliations:** 1 Department of Forest Ecology and Management, Swedish University of Agriculture Sciences, SE-901 83 Umeå, Sweden; 2 Department of Ecology, Swedish University of Agriculture Sciences, SE-750 07 Uppsala, Sweden; 3 Umeå Plant Science Center, Department of Forest Genetics and Plant Physiology, Swedish University of Agricultural Sciences, SE-901 83 Umeå, Sweden; 4 Department of Ecology and Environmental Science, Umeå University, SE-901 87 Umeå, Sweden

## Abstract

In nitrogen (N) limited boreal forests, N enrichment can impact litter decomposition by affecting litter quality and by changing the soil environment where litter decomposes. We investigated the importance of litter quality and soil factors on litter decomposition using a 2-year reciprocal transplant experiment for *Picea abies* needle litter, derived from plots subjected to 17 years of N addition, including control, low and high N treatments (ambient, 12.5 and 50 kg N ha^−1^ yr^−1^, respectively). Our data show that changes in soil factors were the main pathway through which N impacted litter decomposition, with rates reduced by ~15% when placed in high N relative to control plots, regardless of litter origin. Litter decomposition was correlated to soil microbiota, with *Picea abies* litter decomposition positively correlated with gram negative and fungal functional groups. Our results suggest that previous findings of increase soil C accumulation in response to N deposition is likely to occur as a result of changes in soil microbiota rather than altered litter quality.

## Introduction

Anthropogenic activities have increased global emissions of reactive nitrogen (N_r_), which have resulted in higher rates of atmospheric N_r_ deposition^1^. Net primary production in boreal and temperate forests is often limited by N availability^2–5^, primarily because biological N_2_ fixation and soil mineralization rates are very low^6, 7^. Thus, there has been substantial speculation and interest in whether atmospheric N_r_ deposition could promote C uptake in these N-limited forest environments. There is increasing evidence that N_r_ deposition enhances carbon (C) pools in both vegetation and soils in N-limited ecosystems. While enhanced aboveground biomass in response to N_r_ deposition is fairly easy to explain (*i.e*. growth is enhanced because it is limited by N), it remains unclear why soil C stocks increase in response to N. Soil C pools are of particular interest in northern forests because they are often larger and more stable than C in standing vegetation (*e.g*. 3–6 fold greater in boreal forests)^8^. Thus, understanding why soil C accumulates in response to N is important for understanding the forest ecosystem long-term capacity to take up and store C^5^, and thus to mitigate increasing anthropogenic carbon dioxide (CO_2_) emissions.

Litter decomposition is one of the key processes that influences soil C accumulation in forests. Nitrogen deposition can impact litter decomposition by altering the quality of litter *per se*, as a result of an increase of N plant uptake^9, 10^. It is well established that plant litters having high C:N, lignin:N or lignin:cellulose ratios are more decay resistant^11, 12^, thus any shift in litter stoichiometry in response to N enrichment may affect litter decomposition^13^. In addition to litter quality changes, N enrichment can also impact litter decomposition by altering the soil environment where litter decomposes. For example, N may change soil biological activity by altering the total microbial biomass, relative abundance of fungi *versus* bacteria, or their enzymatic activities^2, 14^. A negative impact of N on soil biota (*e.g*. shift in soil biota composition, alteration in microbial activity) could thereby decrease litter decomposition and lead to enhanced soil C accumulation rates^15, 16^. Thus, anthropogenic N has the potential to simultaneously impact both litter quality and soil biota, however, the relative impact of these responses on plant litter decomposition are poorly understood, and have seldom been investigated in long-term experiments where realistic atmospheric N deposition rates have been simulated.

In this study, we utilized a 17-year N addition experiment in the boreal zone of Northern Sweden. We set up a 2-year reciprocal transplantation experiment to investigate how litter decomposition of *Picea abies*, a typical boreal coniferous tree and largest source of litter at our study site, responded to chronic N_r_ enrichment. Specifically, we sought to understand whether some specific soil properties and/or litter quality properties served as main drivers of litter decomposition in response to anthropogenic N enrichment. We used a long-term replicated N addition experiment consisting of control plots, and two N addition levels, 12.5 (low N treatment) and 50 kg N ha^−1^ yr^−1^ (high N treatment)^17^. The low N treatment is quantitatively similar to upper level N_r_ deposition rates in the boreal region^18^ and therefore, our study system serves as a useful tool to investigate the mechanisms through which N_r_ deposition alters litter decomposition in the boreal region. We tested the following hypotheses: (i) That potential changes in litter quality caused by N enrichment will lead to reduced decomposition rates of the most abundant evergreen species (*P. abies*) in our study system, and (ii) that N enrichment caused changes in soil biota (e.g. reduced total and fungal biomasses) will lead to reduced decomposition rates. By testing these hypotheses together, our study provides a rare opportunity to separate the relative effect of two pathway mechanisms that have been proposed to influence soil C accumulation in N limited northern forest environments^2, 4^.

## Results

**Figure 1:**
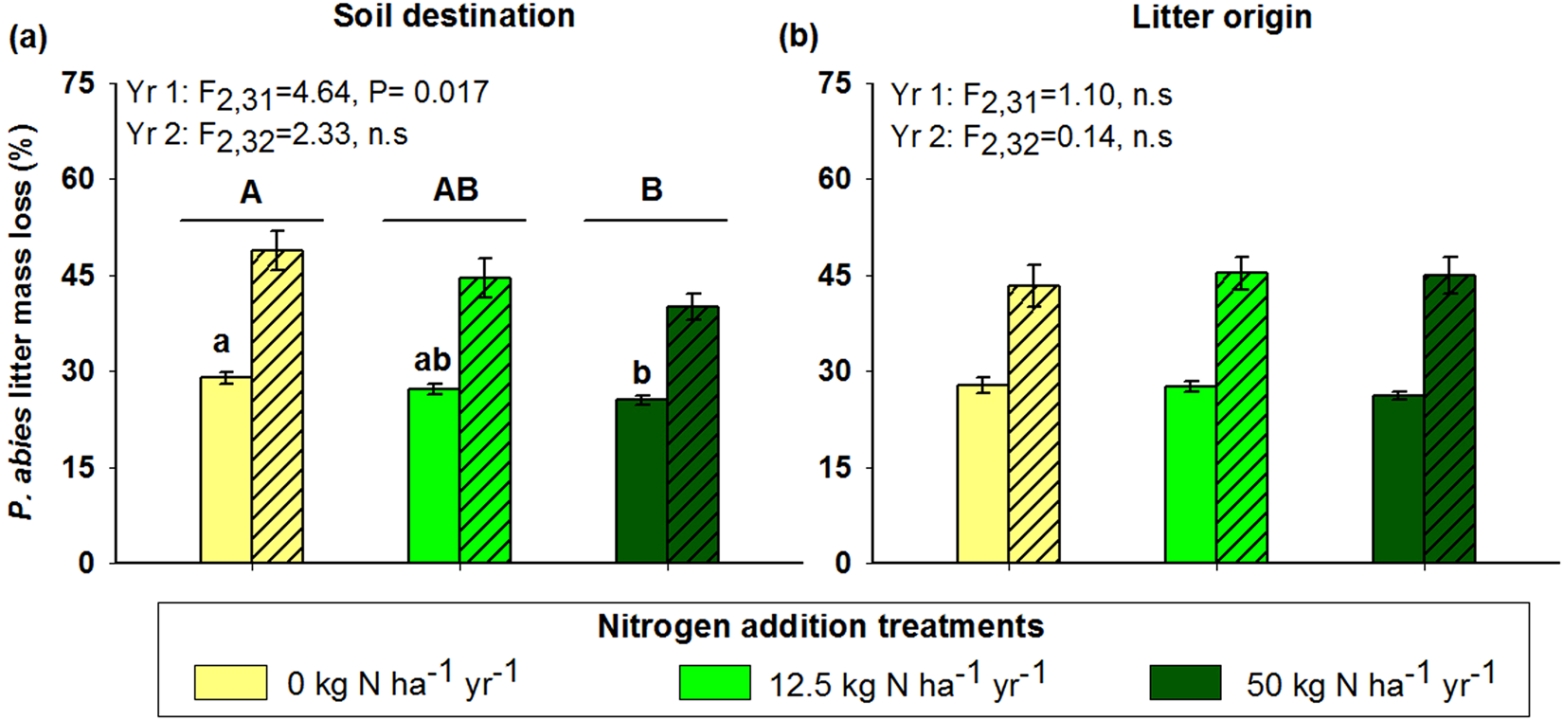
The mean (±SE) mass loss of *Picea abies* litter after decomposing for one (open bars) or two (hatched bars) years in three soil destinations (**a**) or three litter origins (**b**). The soil destinations and litter origins consisted of replicated plots (n = 5) treated with three different N addition levels (0, 12.5 and 50 kg N ha^−1^ yr^−1^). Different capital letters (A or B) on top of each panel groups of two bars are significant differences between treatments regardless time, while different lower case letters (a or b) on top of each group of bars indicate significant differences between treatments determined using Student-Newman-Keuls *post-hoc* tests.

Litter mass loss was significantly affected by the main effects of soil destination and time (Table 1, Fig. 1a). The litter decomposition significantly declined by ~15% and non-significantly declined by ~7% when located in the high N (50 kg N ha^−1^ yr^−1^) and low N (12.5 kg N ha^−1^ yr^−1^) plots relative to the control plots, respectively, regardless of the litter origin (Fig. 1a; Table 1). The soil destination effect was mainly due to a significant decline of the litter mass loss during the first year of decomposition, where the litter decomposed significantly slower by ~11% and non-significantly slower by ~6% in the high N and low N treatment plots relative to the control, respectively. There was a significant time effect because the mass loss was more important during the first year compared to the second year of decomposition. Litter origin had no significant effect on litter mass loss for either sampling time (Table 1, Fig. 1b).

**Table 1:** The F-values, degrees of freedom (df), and P-values from a repeated-measures ANOVA evaluating the effect of soil destination (S), litter origin (L), sampling time (T) and their interactive effects, on litter mass loss across three simulated chronic N deposition treatments (0, 12.5, and 50 kg N ha^−1^ yr^−1^).

	Litter mass loss		
	F-value	df	P-value
Soil destination (S)	3.85	2	**0.029**
Litter origin (L)	0.10	2	0.902
Time (T)	93.53	1	**<0.001**
S × L	0.35	4	0.842
S × T	0.71	2	0.496
L × T	0.28	2	0.757
S × L × T	0.057	4	0.994

Values in bold indicate statistical significance at P < 0.05.

**Figure 2:**
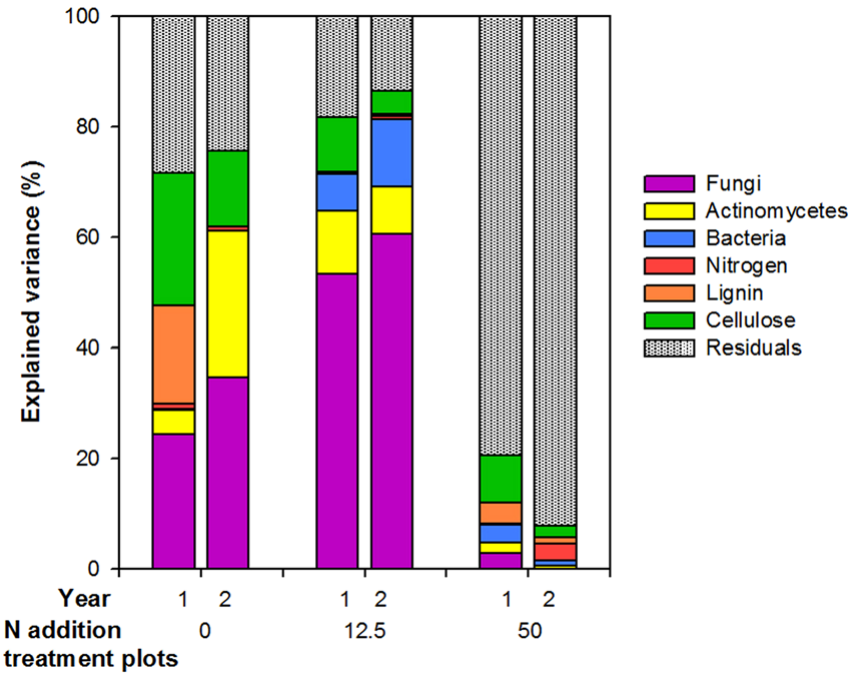
Variance partitioning (%) in litter decomposed in control, low N (12.5 kg N ha^−1^ yr^−1^) and high N (50 kg N ha^−1^ yr^−1^) treated plots during one and two years, explained by soil biota factors: fungal, actinomycetes, bacterial PLFAs and litter quality factors: % nitrogen, % lignin and % cellulose.

Variation partitioning analysis showed that the variation of litter decomposed in the control plots was mainly explained by fungal (24–34%) and actinomycetes (4–26%) PLFA biomasses, as well as the litter’s % lignin (0–18%) and % cellulose (13–24%) (Fig. 2). The variation of litter decomposed in the low N treatment plots was mainly explained by fungal (53–61%), actinomycetes (9–12%) and bacterial (0–12%) PLFA biomasses as well as the litter’s % cellulose (4–10%). The % N factor explained only a small percentage of the variation in litter decomposed in both control and low N plots. The variation of litter decomposed in the high N treatment plots was poorly explained by the soil biota (1–8%) and litter quality (6–13%) factors.

**Figure 3:**
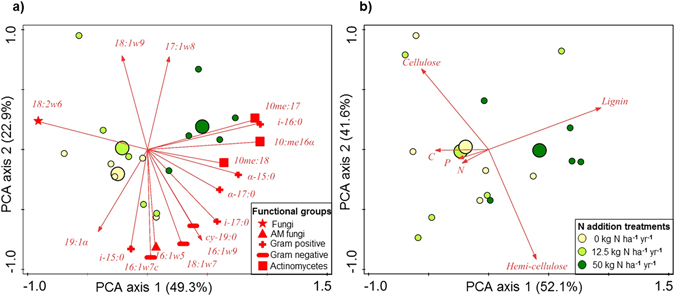
Principal component analysis (PCA) of phospholipid fatty acids (PLFA) microbial markers (**a**) and litter quality parameters (**b**). In both sub-panels circles depict N addition treatments (yellow = control; light green = low N, 12.5 kg N ha^−1^ yr^−1^; dark green = high N, 50 kg N ha^−1^ yr^−1^). Small circles indicate individual plots, and large circles indicate the average PCA position for each treatment. In sub-panel a, only the best fitting microbial markers are represented.

The RDA analysis using Monte Carlo permutation tests showed that significant differences in PLFA markers were present among the N treatments (P-1^st^ axis = 0.012; P-2^nd^ axis = 0.007), while litter parameters showed no significant differences among N addition treatments (P-1^st^ axis = 0.096; P-2^nd^ axis = 0.159). The PCA analysis describing the soil microbial PLFA marker data set had 49.3% and 22.9% of the total variation explained by the first and second axis, respectively (Fig. 3a). The primary drivers of the first PCA axis were the fungal PLFA markers (18:2 ɷ6), the actinomycete PLFA markers (10me16:0, 10me17:0, 10me18:0) and the gram positive PLFA markers (i-16:0, α-15:0, α-17:0). Meanwhile, the second axis was driven by the gram negative PLFA markers (16:1ɷ7, 18:1ɷ7 and cy-19:0), the arbuscular fungal PLFA marker (16:1ɷ5), and general microbial markers (18:1ɷ9 and 17:1ɷ8). The fungal functional group was negatively related to the control and low N plots, while the gram positive and actinomycete functional groups were positively related to the high N plots. The PCA analysis describing the litter parameter data set had 52.1% and 41.6% of the total variation explained by the first and second axis, respectively (Fig. 3b). The primary drivers of the first PCA axis was the % lignin, whereas the % C, N and P were poorly related to the first axis. Meanwhile, the second axis was driven by the % cellulose and hemi-cellulose. The control and low N plots were overlapping on the biplot-PCA (Fig. 3b), while the % lignin was positively related to the high N plots.

**Figure 4:**
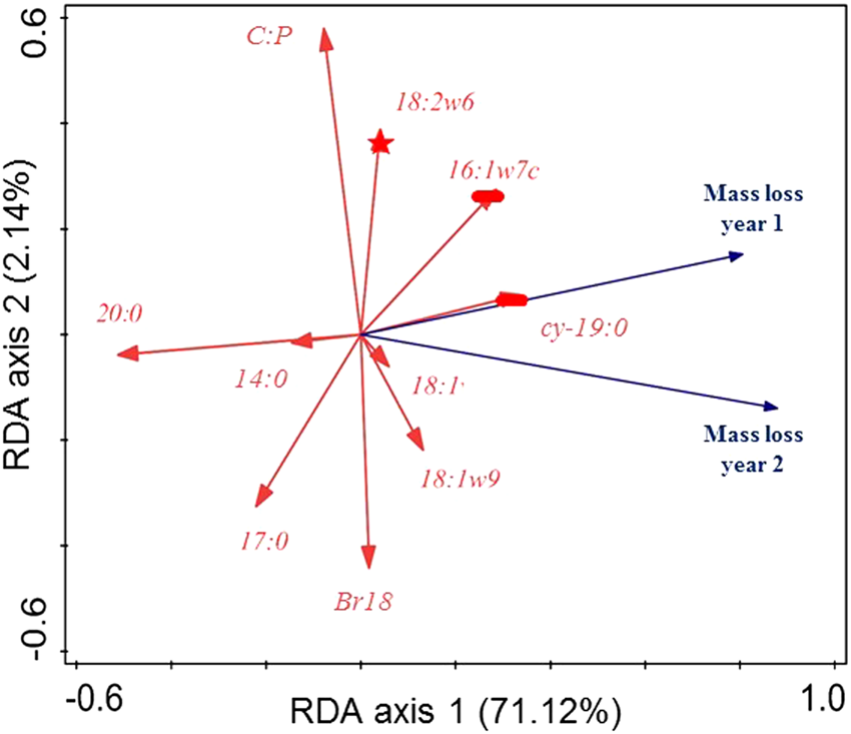
Redundancy analysis (RDA) of 1 and 2 year litter mass loss during a decomposition assay, phospholipid fatty acid (PLFA) microbial markers, and litter quality parameters as affected by three N addition treatments (control, 12.5, and 50 kg N ha-1 yr-1). For clarity, only the best fitting explanatory variables are represented. Gram negative functional group are represented by a bar and fungal functional group by a star.

Litter mass loss was mainly driven by microbial PLFA markers than litter quality parameters (Fig. 4, Table S1). The primary drivers of the first RDA axis (P-1^st^ axis = 0.002) were the gram negative PLFA marker (cy-19:0) and general microbial PLFA marker (20:0 and 14:0), while the gram negative PLFA marker (16:1ɷ7c) and the general microbial markers (18:1ɷ9 and 17:0) drove both the first and second RDA axes. Meanwhile, the second axis (P-1^st^ axis = 0.002) mostly driven by the fungal PLFA marker (18:2ɷ6), the C:P litter ratio and the general microbial marker (br18:0). The first year of litter mass loss was most positively associated with gram negative PLFA markers (cy-19:0 and 16:1ɷ7c), the fungal PLFA marker (18:2ɷ6), general microbial PLFA marker (18:1 and 18:1ɷ9) and negatively correlated with the general microbial PLFA markers (20:0, 17:0 and 14:0). The second year of litter mass loss was most positively associated with general microbial PLFA markers (18:1, br18:0 and 18:1ɷ9), gram negative PLFA markers (cy-19:0 and 16:1ɷ7c) and negatively correlated with several other general microbial PLFA markers (20:0, 17:0 and 14:0) and the C:P litter ratio. In summary, both litter mass loss of year 1 and 2 were associated with numerous soil microbial PLFA parameters, and were rarely associated with litter quality parameters.

## Discussion

Our aim was to investigate how litter decomposition of a common coniferous tree species in boreal forests responded to anthropogenic N enrichment, and to distinguish the relative importance of altered soil microbiota and litter quality factors as drivers of litter decomposition. To our knowledge, our study system is the longest running experiment to simulate realistic levels of atmospheric N deposition in the boreal biome (≤12.5 kg N ha^−1^ yr^−1^)^18, 19^, providing us a unique opportunity to explore the mechanisms through which chronic N deposition affects litter decomposition, and thus soil C sequestration.

Contrary to our first hypothesis, decomposition of *P. abies* litter was unresponsive to litter origin, suggesting that N induced changes in litter chemistry were either too small to have a large impact on decomposition, or that litter quality in general had a minor impact on decomposition relative to other factors. In support of this point, multivariate analyses (*i.e*. RDA, PCA) showed no clear differences in litter parameters among N addition treatments (*i.e*. for litter origin); and nor any association between litter parameters and litter mass loss during the first and second year of decomposition. Despite litter origin having no significant impact on litter decomposition, the variation in the *P. abies* litter mass loss induced by the N addition treatments was partly explained by random variation in the initial cellulose and lignin content of the litter. Our findings are consistent with lignin and its associated ratios (*e.g*. lignin:cellulose) that have been shown to be good predictors of litter decomposition rates as lignin is a more recalcitrant plant compound than cellulose^12, 13^. While litter quality parameters *per se* were important in predicting random variation in litter decomposition, altered litter quality was not a significant mechanisms through which N addition caused a shift in litter decomposition rates in our study system.

In support of our second hypothesis, soil destination showed a significant main effect on litter decomposition, whereby litter placed in the high N plots decomposed significantly slower (i.e. 15%) relative to the control plots, while the litter placed in the low N plots decomposed non-significantly slower by 7%. This effect was significant on average across sampling times, but was particularly strong for *P. abies* litter after one year of decomposition. Further, support for our second hypothesis was provided by significant relationships between litter mass loss and soil biota. Interestingly, litter mass loss was positively correlated with gram negative and fungal biomasses during the first year of decomposition. Further, the variation in the litter mass loss induced by the N addition treatments was mainly explained by the soil fungal biomass when the litter decomposed in the control and the low N plots. This latter point was also supported by an association between the soil fungal functional group and the control and low N addition treatments in the PCA, while all bacterial functional groups were associated with the high N addition treatment. In contrast, the variation in the litter mass loss induced by the high N addition treatment was poorly explained by the soil biota groups significant in our study. This could be due to other factors that has not been measured *e.g*. changes in plant community structure, with a decline of bryophyte and ericaceous shrubs cover^20–22^ as well as an increase of Norway spruce tree volume^23^ in response to long-term 50 kg N ha^−1^ yr^−1^ that may change the microclimate where the litter decomposed.

Our data are consistent with soil fungi being well known to dominate the soil microbial community compared to bacteria in boreal forest ecosystems^24, 25^. Several studies have shown a decrease in fungal biomass and enzymatic activities in response to N fertilization^2, 14, 26^. The decomposition of coniferous plant litter requires an arsenal of both hydrolytic and oxidative enzymes to degrade litter tissue compounds (*e.g*. lignin, cellulose and hemi-cellulose)^27, 28^, thus any shift or decline in soil fungi may explain our observed decline in litter decomposition with N addition, as well as the reduced contribution of fungi in explaining variation in decomposition within the high N plots.

While our study evaluated litter decomposition during only 2 years, the litter achieved nearly 50% mass loss during this period. Norway spruce litter decomposition has been estimated to reach a limit value (*i.e*. the point at which the mass loss approaches a final value that follow an asymptotic function) of *ca*. 61–68% of the mass loss in boreal ecosystems^28, 29^. Thus, our 2-year litter decomposition experiment covered approximately 66–74% of the limit value and likely included some later stage decomposition in addition to the entire early stages of decomposition. Although our study was not able to explicitly inform on the later stages of decomposition, some work has suggested that nutrient enrichment effects on microbial community composition can effect both early and late stages of decomposition^30, 31^. For instance, Hobbie *et al*.^30^ showed that early litter decomposition stages were associated with an increase of microbial enzymatic activity as well as gram positive and negative abundances in response to N addition while they observed a decline of both microbial enzymatic activity and gram positive and negative abundances during later decomposition stages in a deciduous forest.

Our results have implications for understanding how N deposition can impact C accumulation in boreal soils^2, 5^, which are known to serve as an important sink in the global C cycle. First, we show that N enrichment effects on litter quality did not cause discernable changes in litter decomposition; whereas, changes in soil microbiota strongly influenced early stage decomposition rates, regardless of any effect that N may have had on litter quality. Secondly, our data suggest that decomposition decline in response to N deposition, which correspond to decreases of litter decomposition by 2.14% and 4.34% per year in the low and high N addition treatment relative to the control, respectively. Studies conducted in the same experimental site showed that 16 years of N addition increased the organic soil C in both N addition treatments^2^, while contemporary aboveground litter inputs were little affected by N addition^20^. As such, our identification of one of the key mechanisms through which anthropogenic N deposition impact litter decomposition may improve current modelling of ecosystem C dynamics in response to anthropogenic N enrichment. Further, our findings suggest that future research focused on soil C responses to N should investigate further how N alters microbial community composition and biomass, and the enzymatic processes they drive.

## Methods

This study was performed at Svartberget Experimental Forest (64°14′N, 19°46′E) in the middle boreal zone, near Vindeln, Sweden^32^. The forest site consists of a late successional (~120 years old) closed canopy Norway spruce forest (*Picea Abies* (L.) Karst.). The understory vegetation is dominated by the ericaceous species, namely *Vaccinium myrtillus* L^3^, and a moss layer consisting of *Hylocomium splendens* (Hedw.) B.S.G, *Pleurozium schreberi* (Bird), and *Ptilium crista-castrensis* (Hedw.). Soils at the site are podzols formed from glacial till^33^. The background atmospheric N_r_ deposition rate in the area is approximately 2 kg N ha^−1^ yr^−1^ ^34^ and the mean annual precipitation is 583 mm. In 1996, a long-term N addition experiment was set up at the site, consisting of a randomized-block design of 0.5 ha plots (n = 5) assigned to one of three N addition treatments: 0 (control), 12.5 (low N treatment) or 50 kg N ha^−1^ yr^−1^ (high N treatment)^17, 35^. The low N treatment was chosen to simulate the upper level N deposition rate in the boreal zone^18, 19^, while the high N treatment has been chosen as a useful comparison with many previous boreal forest fertilization experiments^4^. The N addition treatments have been applied since 1996 by manually spreading ammonium nitrate granules each year after snow melt (*i.e*. May).

Using litter traps, we collected *Picea abies* needle litter monthly between the start of June 2012 and the end of May 2013 in each plot. Norway spruce litter was chosen as main subject of the study because a previous study showed it was the most abundant litter category in our study system^20^. The collected litter was air-dried and thoroughly homogenized. Litter Subsamples were used to estimate the moisture content as well to characterize the litter chemistry (*i.e.* % C, % N, % P, % lignin, % cellulose, % hemi-cellulose). *Picea abies* litter was placed in nylon cloth material (8 × 7 cm) with mesh size of 0.3 × 0.1 mm, as done in numerous other studies^36, 37^. The litter bags contained a dry mass of 2.00 g dry weight of *Picea abies* litter.

In autumn 2013 (17 years after the start of N treatments), in order to study whether litter quality and/or soil environment mediated by N addition contribute to aboveground litter decomposition, a reciprocal transplant experiment was set up with *P. abies* litter bags originated from each N treatment (control, low and high N treatments). Two litter bags were then placed back in the plot they originated from, as well as within each of the other N treatments within the same block. The litter bags were pinned to the forest floor with plastic sticks in October, and were collected after one and two years of incubation (*i.e*. autumn 2014 and 2015). This design resulted in a total of 90 litter bags (3 litter origins × 3 soil destinations × 2 durations). After collection, the litter bags were oven dried at 60 °C for 48 h to prevent further decomposition and weighted. Litter mass loss for each species was expressed as relative mass loss (% mass loss).

Soil biota characteristics were derived from a previous study in our experimental site^2^. Soil biota of each treatment were analyzed from the humus layer using a modified method of Bligh and Dyer on microbial phospholipid fatty acids method (PLFA)^38–40^. Soil microbial PLFA markers were allocated to functional groups: fungal (*i.e.* correlated to ectomycorrhizae), AM fungi (*i.e.* arbuscular mycorrhizae), bacterial, actinomycete, gram positive and gram negative following the nomenclature described in Frostegård *et al*. (2010). Litter parameters such as plant C and N concentrations were analyzed by dry combustion using an elemental analyzer (LECO TruSpec CN analyzer; St. Joseph, MI, USA)^2, 20^, while total P content of plant litter was analyzed by acid digestion (Auto Analyzer III Spectrophotometer, Omniprocess, Germany). Lignin, cellulose and hemi-cellulose litter contents were also analyzed by acid digestion and calcination all performed by the Soil, Water and Plant Testing Laboratory, Colorado State University, USA^20^.

In order to describe both soil microbial and litter parameter data sets, we first performed a detrended correspondence analysis (DCA) to be able to choose between linear and unimodal methods. The gradient length were 0.2 SD-units and 1.1 SD-units, respectively, we thus used linear methods. We first performed partial redundancy analyses (RDA) with block as a random factor with Euclidean distance matrices using a Monte Carlo Permutation test (n = 999, α = 0.05) in order to determine whether multivariate differences in the overall PLFA signatures and in the litter parameter data sets (*i.e*. dependent variables) occurred in response to the N addition treatments (*i.e*. independent variable). We then performed PCA analyses (type I scaling-distance biplot) to summarize both soil microbiota and litter data sets. Microbial community structure was characterized by performing a PCA on the log-transformed PLFA biomarkers (relative proportion as % of the total), while litter parameters were log transformed. We also performed a variation partitioning analysis to determine the relative importance of soil microbiota biomass (*i.e*. fungi, actinomycetes, bacteria) and litter quality parameters (*i.e*. lignin, cellulose and N) on *P.abies* litter decomposition. The percentage of each variable was determined using the pamer.fnc function within the LMERConvenienceFunctions R package^41^. To determine how soil microbial community and litter chemistry were driven the litter mass loss during year 1 and 2, we used an RDA with litter mass loss from year 1 and year 2 as dependent variables and microbial and litter parameters as independent variables (type II scaling-correlation plot). The explanatory variables were selected by forward selection and retained when significant amount of variance was explained (α < 0.05)^42^. All multivariate analyses examining relationships between environmental and response variables were interpreted according to Šmilauer & Lepš (2014)^43^.

All response variables were first tested for normality using Shapiro-Wilk test prior to statistical analyses. Homoscedasticity was testing using Levine´s test on the residuals of the statistical model. Litter mass loss was analyzed using a repeated-measures mixed model in which soil destination, litter origin and time were used as fixed factors (α = 0.05). Block was used as a random factor. When significant differences between N addition levels were detected (α = 0.05), *post hoc* pairwise comparisons between treatments were conducted using the Student-Keuls test. Statistical analyses were performed using SPSS (v. 22.0) and R (v. 3.2.3), while multivariate analyses were performed with CANOCO (v. 5.0).

## Electronic supplementary material

Supplement table 1
