# Supplementary material for: Nitrogen enrichment impacts on boreal litter decomposition are driven by changes in soil microbiota rather than litter quality
Source: Sci Rep. 2017 Jun 22;7:4083. doi: 10.1038/s41598-017-04523-w (PMC5481386; doi:10.1038/s41598-017-04523-w)
Supplement: Supplementary file 1 — Supplement table 1 [file 41598_2017_4523_MOESM1_ESM.doc]

**Nitrogen enrichment impacts on boreal litter decomposition are driven by changes in soil microbiota rather than litter quality**

Nadia I. Maaroufi, Annika Nordin, Kristin Palmqvist, and Michael J. Gundale

Supplement table

Results from the forward selection of the explanatory variables found to have a significant effects (α< 0.05) and included in the redundancy analysis (RDA) of the microbial phospholipid fatty acids (PLFAs) and the litter parameters as predictors of the litter mass loss during the first and second year of decomposition. C:P= carbon: phosphorus ratio.

|  | Litter mass loss | | |
| --- | --- | --- | --- |
| Explanatory variable | Explained variation (%) | Pseudo F | p-value |
| 20:0 | 23.1 | 9.6 | 0.004 |
| 18:1 | 11.2 | 5.2 | 0.012 |
| 14:0 | 12.4 | 6.5 | 0.014 |
| 18:1ɷ9 | 9.4 | 5.4 | 0.020 |
| 16:1ɷ7c | 9.4 | 6.2 | 0.006 |
| cy19:0 | 5.2 | 3.6 | 0.044 |
| 18:2ɷ6 | 4.2 | 3.1 | 0.048 |
| 17:0 | 6.1 | 5.0 | 0.014 |
| C:P litter ratio | 5.1 | 4.7 | 0.030 |
| Br18 | 4.4 | 4.4 | 0.028 |
